# Strong Quantum Confinement of 2D Excitons in an Engineered 1D Potential Induced by Proximal Ferroelectric Domain Walls

**DOI:** 10.1021/acs.nanolett.5c02438

**Published:** 2025-08-12

**Authors:** Pedro Soubelet, Yao Tong, Asier Astaburuaga Hernandez, Peirui Ji, Katia Gallo, Andreas V. Stier, Jonathan J. Finley

**Affiliations:** † Walter Schottky Institut and TUM School of Natural Sciences, Technische Universität München, Am Coulombwall 4, 85748 Garching, Germany; ‡ Department of Physics, KTH Royal Institute of Technology, Roslagstullsbacken 21, Stockholm SE-10691, Sweden

**Keywords:** two-dimensional semiconductors, transition-metal dichalcogenides, periodically poled lithium niobate, ferroelectric domain walls, one-dimensional exciton confinement

## Abstract

We investigate the confinement of neutral excitons in a one-dimensional (1D) potential engineered by proximizing hexagonal boron nitride (hBN)-encapsulated monolayer MoSe_2_ to ferroelectric domain walls (DWs) in periodically poled LiNbO_3_. Our device exploits the nanometer scale in-plane electric field gradient at the DW to induce dipolar exciton confinement via the DC Stark effect. Spatially resolved photoluminescence spectroscopy reveals the emergence of narrow emission lines redshifted from the MoSe_2_ neutral exciton by up to ∼100 meV, depending on the sample structure. The spatial distribution, excitation energy response, and polarization properties of the emission are consistent with the signatures of 1D-confined excitons. The large electric-field gradients accessible via proximal ferroelectric systems open up new avenues for the creation of robust quantum-confined excitons in atomically thin materials and their heterostructures.

## Graphical abstract

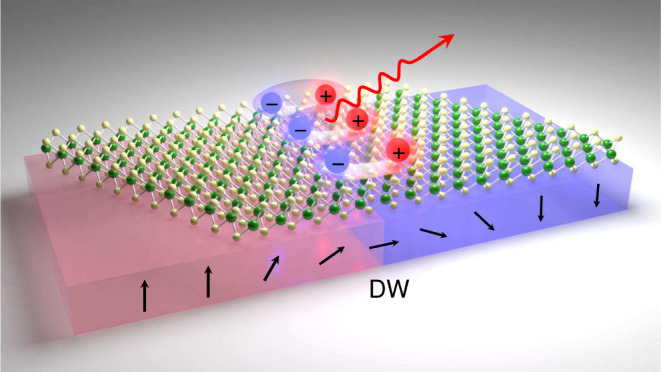

The confinement of particles to length scales comparable to their de Broglie wavelength leads to the quantization of their motional ground states.^1^ When the thermal energy of the system, *E*_T_ ≃ *k*_B_ *T*, falls below the separation between these states, interaction strength and coupling of quantum degrees of freedom to environmental excitations become strongly modified. Functional quantum technologies hinge on the precise manipulation of particles in this quantum regime.^2–5^ Furthermore, interactions are significantly enhanced by reducing the system dimensionality,^6–16^ enabling the exploration of emergent quantum phases.^17–23^ In this context, monolayer transition-metal dichalcogenides (1L-TMDs) have gained attention due to their inherent two-dimensional (2D) confinement. These materials are of direct band gap at the *K*/*K*′ points of their hexagonal Brillouin zone,^24–26^ where interband optical transitions form tightly bound excitons.^27–31^ While excitons in 1L-TMDs couple strongly to light, achieving motional quantization remains challenging due to their heavy masses and small Bohr radii.^30,31^ For instance, achieving an energy splitting of ℏω ≳ 1 meV between discrete ground states requires confinement length scales of $l_{n}=\sqrt{\frac{\hbar }{m_{X}\omega }}≲10$ nm for an exciton mass (*m*_X_) on the order of the free electron mass (*m*_e_).^32^

The manipulation of excitons in 2D-semiconductor materials has largely centered around approaches such as moiré potential engineering,^7,22,33–36^ strain engineering,^3,37–41^ and tuning of the local dielectric environment^28,42–47^ to create interlayer junctions and trapping potentials. Furthermore, point defects have been created using electron- and ion-beam irradiation that create optically active centers.^48,49^ The use of partially overlapping gates^32,50,51^ has recently been shown to facilitate the generation of one-dimensional (1D) exciton states^32,50^ and enable control of exciton wave functions.^51^ This method utilizes the DC Stark shift induced by an in-plane electric field,^52,53^ in combination with the formation of lateral p–i–n junctions, where the p and n regions are defined by the gate arrangement to create a confinement potential.^32,50,51,54^ This technique offers the advantage of in situ tuning of the confinement potential from a continuum of 2D exciton states to the 1D regime, where excitons are confined within potential traps ∼5 meV deep.^32,50^ Because the total confinement potential arises from the interplay of the DC Stark shift and repulsive Coulomb interaction,^32^ the 1D exciton states are not completely decoupled from the 2D counterpart.

In this work, we demonstrate how nanometer-scale ferroelectric domain boundaries in lithium niobate (LiNbO_3_)^55–57^ are used to induce strong 1D confinement of neutral excitons in 1L-MoSe_2_, dominated by the Stark effect. LiNbO_3_ is a versatile ferroelectric material that can be integrated on oxide sacrificial layers using CMOS-compatible processes to produce low-loss waveguides,^58,59^ making it highly suitable for integrated optoelectronics and photonics.^60–62^ Due to its strong ferroelectric properties, with spontaneous polarization of ∼70 μC cm^–2^ and a Curie temperature exceeding 1400 K,^63^ LiNbO_3_ exhibits a robust polarization and electric field that persists across a wide temperature range, making it suitable for applications even at cryogenic temperatures.^64^ Furthermore, periodically poled lithium niobate (PPLN) exhibits large surface charge densities within individual ferroelectric domains and atomically sharp Neél-type domain walls (DWs) between domains.^56,63,65,66^ Previously,^67^ we demonstrated that the in-plane electric field (*E*_x_) at the DWs establishes a lateral p–n junction-like potential landscape in 1L-TMDs deposited on top of such domains. This potential induces 2D exciton drift and dissociation near the DW. The magnitude of *E*_x_ reaches up to ∼400 V μm^–1^ and is localized to length scales of a few nanometers, far beyond what can be achieved using metallic contacts and suggesting that DWs can serve as quantum traps for excitons via the DC Stark effect.^52,53^ To validate this, we performed spatially resolved micro-photoluminescence (μPL), photoluminescence (PL)-excitation (PLE), and polarization-resolved spectroscopies on hexagonal boron nitride (hBN)-encapsulated 1L-MoSe_2_ samples transferred onto a PPLN substrate. Our results reveal the formation of 1D exciton states localized at the DW, with narrow emission lines redshifted by up to ∼100 meV from the MoSe_2_ neutral exciton, depending on the sample structure. These observations are consistent with exciton center-of-mass (COM) confinement over length scales as small as ∼3 nm. The absence of interactions with surrounding free charges, combined with thermal robustness observed through temperature-dependent PL experiments, suggests that proximal electric fields from ferroelectric DWs may be highly interesting for exploring strongly correlated exciton states.^23^

## Results and Discussion

Figure 1a shows a schematic of a van der Waals layered device, representative of the devices used in this study. The detailed structures of each device, along with the fabrication processes, are presented in Supporting Information (SI) Notes I and IV.^68^ The designs exploit the electric field at ferroelectric Néel-type DWs in LiNbO_3_,^65,69^ depicted in Figure 1a by the rotating arrows. The large and nanometer scale in-plane component of this electric field (*E*_x_) polarizes the TMD neutral exciton, resulting in a local reduction of the exciton transition energy and providing an attractive potential *V*_Stark_ within 1L-MoSe_2_ directly above the DW due to the DC Stark effect,^52^

1 $$V_{\mathrm{Stark}}=-\frac{1}{2}\alpha {E_{x}}^{2}$$

where α = 6.5 nm^2^ V^–2^ ^52^ is the in-plane exciton polarizability of MoSe_2_. To estimate the in-plane electric field length scale and confinement potential, we calculated the electric field in the 1L-MoSe2 plane as a function of the bottom hBN thickness using finite-element simulations. The resulting potentials are presented in Figure 1b for bottom hBN thicknesses varying from 0 to 10 nm, highlighting the possibility to tune *V*_Stark_ by choosing the appropriate bottom hBN thickness. Further details on the simulations of the electric field are provided in SI Note II.^68^ To tune the electronic landscape in the TMD, 1L-MoSe_2_ was encapsulated in thin flakes of hBN and a top gate was incorporated using a thin graphite flake. The ohmic contact to 1L-MoSe_2_ was oriented across the DW to ensure that all sample regions are grounded, regardless of the junction potential.

**Figure 1:**
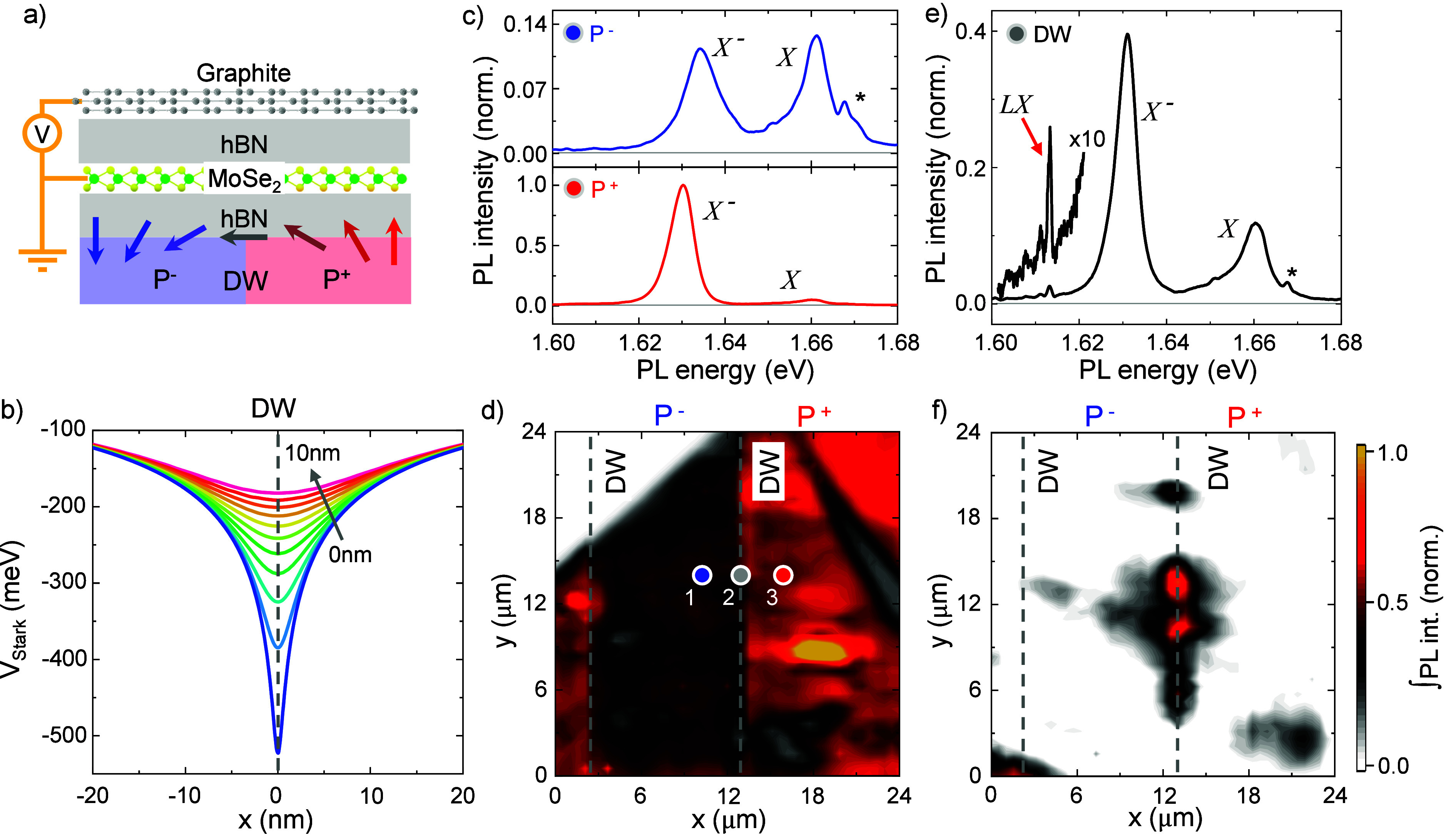
Sample description and emission signatures of 1D-confined excitons. (a) Schematic of the hBN-encapsulated 1L-MoSe_2_ straddling DW boundaries in PPLN. The red-to-blue arrows indicate the direction of the PPLN electric field near the DW, which generates a 1D trapping potential (*V*_Stark_). (b) Calculated 1D trapping potential in the plane of 1L-MoSe_2_ for a variety of bottom hBN thicknesses from 0 to 10 nm. (c) μPL spectra recorded at the P^–^ and P^+^ domains (top and bottom panels, respectively) showing the *X* and *X* ^–^ emission. The sample was excited using a continuous-wave optical power *P*_ex_ = 1 μW, and the excitation photon energy was set to *E*_ex_ = 1.722 eV, ∼60 meV above the 1L-MoSe_2_ *X*. Features marked with an asterisk (*) correspond to Raman lines from the substrate. (d) False color map showing the integrated PL across the sample. The spatial modulation reveals the underlying PPLN domains. Spots 1 and 3 mark the sites in which the spectra in panel (c) were acquired. (e) μPL spectra recorded at the DW (spot 2 in part d). In addition to the *X* and *X* ^–^ emission, the spectra feature narrow emission lines (*LX*s) redshifted by ∼50 meV from *X*. (f) False color map showing the integrated background-subtracted PL intensity of the *LX*.

We begin our investigation by presenting spatially resolved low-temperature (*T* = 4.2K) PL spectroscopy on a sample with a 8-nm-thick bottom hBN (Sample 1). Unless otherwise specified, all experiments were conducted with both the top gate and the sample grounded. Figure 1c shows selected PL spectra corresponding to two distinct domains with opposite out-of-plane ferroelectric polarization (P^–^ and P^+^, respectively).^67^ Experimental details are given in SI Note I.^68^ The main difference between these spectra originates from modulation of the free charge density across the domains. Features marked with an asterisk (*) correspond to Raman lines from the substrate (see SI Note III for details). On the basis of the trion binding energy,^70^ we estimate a negative free charge density of $n_{P^{-}}=\left(0.42\pm 0.03\right)\times 10^{12}$ cm^–2^ and $n_{P^{+}}=\left(0.98\pm 0.03\right)\times 10^{12}$ cm^–2^ for the P^–^ and P^+^ domains, respectively. For details regarding such estimations and the extraction of spectral positions, see SI Note III.^68^ Figure 1d shows a false-color map of the integrated intensity of the 1L-MoSe_2_ emission across the device. The spectra in Figure 1c were obtained from the locations labeled as “1” (blue dot) and “3” (red dot) in Figure 1d. The PL map thus effectively reveals the ferroelectric domain structure. Based on this spatial modulation, the ferroelectric polarization of each domain was determined in accordance with our previous work.^67^

The key observation is shown in Figure 1e, where we plot the spectrum recorded directly at the central DW (gray dot “2” in Figure 1d) showing narrow emission lines (*LX*s) redshifted by ∼50 meV relative to the neutral exciton (*X*). We follow the spatial distribution of these features by integrating the 1L-MoSe_2_ emission over the *LX* spectral range (1.595–1.615 eV), while subtracting the broad *X* ^–^ emission as the background. The resulting map is shown in Figure 1f, indicating that these narrow lines are macroscopically localized along the central DW (*x* ≃ 12 μm). Notably, the PL map in Figure 1d shows that the DW at *x* ≃ 3 μm is not sharply defined, and consequently no *LX* is observed in Figure 1f. To better understand the distribution of these emission lines along the DW and to distinguish them from other localized emissions, see SI Note IV.^68^ For the remainder of this paper, we demonstrate that the *LX* emission is consistent with the radiative recombination of excitons confined in a 1D quantum trap at the DW.

The *LX* emission lines were observed in several samples. Panels (a)–(c) of Figure 2 summarize low-*T* μPL linescans across the DWs in three samples (details in SI Note IV^68^). In each panel, the DW location is indicated with a gray dashed line. The main difference between samples is the bottom hBN thickness, as indicated. All samples show the neutral exciton *X* and trion (*X* ^–^), and, diffraction limitation at the DW, *LX* emissions with energies relative to *X* of ∼−50, ∼−70, and ∼−120 meV for Samples 1–3, respectively. To the best of our knowledge, such tunability up to 120 meV has not been observed for point defects within MoSe_2_ through the application of neither an electric field nor strain. Moreover, in SI Note V,^68^ we analyze the possibility of strain and show that it is below 0.004%, ruling out its influence on the *LX* emission. Our experiments show that reducing the bottom hBN thickness causes the *LX* emission to redshift relative to *X*, demonstrating tunability of the confinement potential by controlling *E*_x_ in the 1L-MoSe_2_ plane. To validate this interpretation, we resolved the Schrödinger equation for the calculated dipolar potentials (Figure 1b), obtaining the wave functions and eigenenergies of the system (see SI Note V^68^). Figure 2d shows the calculated binding energy of the confined ground state (ψ_0_) and the relative *LX* energy with respect to *X*. The trend agrees with simulations, although the model overestimates the *LX* confinement. Specifically, the calculated binding energy is ∼300 meV higher for thin hBN thicknesses and ∼100 meV higher for hBN thicknesses of >5 nm. This mismatch likely stems from our oversimplified dielectric model for thin hBN layers^71^ and variations of the trapping-induced exciton binding energy, whose detailed study is beyond the scope of this work.

**Figure 2:**
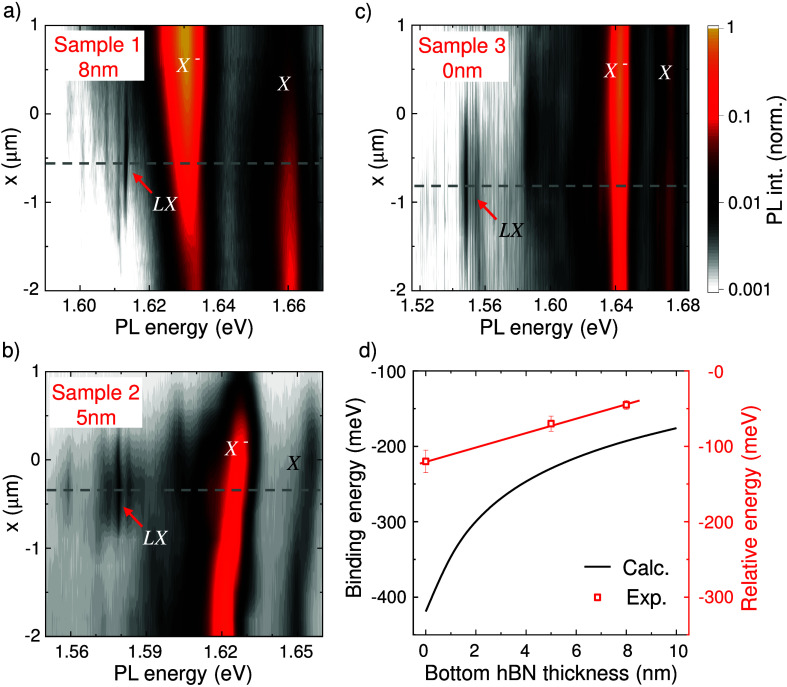
PL experiments across samples with different bottom hBN thicknesses. (a–c) False-color plots of the spatially resolved emission from 1L-MoSe_2_ across the DW for three different samples. Sample 1 is a 1L-MoSe_2_ stacked on top of 8-nm-thick hBN, Sample 2 a 1L-MoSe_2_ stacked on top of 5-nm-thick hBN, and Sample 3 is a 1L-MoSe_2_ directly stacked on top of the PPLN. The gray dotted line marks the DW position. (d) Comparison between the calculated binding energy of the first confined state (ψ_0_) and the experimentally observed localized states (*LX*) energy relative to the *X* energy for the three samples. The red line serves as a guide to the eye.

We continue the discussion by presenting data from Sample 1 (8 nm bottom hBN). In MoSe_2_, localized emission from defects occurs within an energy range similar to that of the observed *LX* emission in this sample.^72^ Because a 1D system exhibits a continuous density of states, in contrast to the discrete energy levels characteristic of localized defects, we conducted power-dependent PL experiments to distinguish between those possibilities. Figure 3a presents selected PL spectra of *LX* emission, resonantly exciting *X* at *E*_ex_ = 1.66 eV, with varying excitation power *P*_ex_ from 7 nW to 200 μW. At very low *P*_ex_, the spectrum consists of a single narrow line (line width ∼400 μeV, *LX*_0_) superimposed on a broad background. With an increase in *P*_ex_, the background develops some structure while maintaining its relative intensity with respect to the *LX*_0_ line. Additionally, a few narrow peaks emerge from the noise floor, blue detuned from *LX*_0_ (e.g., *LX*_1_ and *LX*_2_). These higher-energy narrow emission lines, spaced by ∼3.5 meV, are consistent with the presence of discrete quantized energy levels within the confinement potential. These levels become optically active while increasing their population under higher laser excitation and emerge due to state filling. Figure 3b shows the integrated PL intensity of these features versus *P*_ex_. Notably, the background, *LX*_0_, and *LX*_1_ exhibit the same power-law dependence as *X* ^–^, scaling approximately linearly (*s* = 0.97 ± 0.01).The absence of any saturation over 5 orders of magnitude of *P*_ex_ indicates that the *LX* emission is not quantum-dot-like (0D trapping), as expected in the case of defects in MoSe_2_.^72^

**Figure 3:**
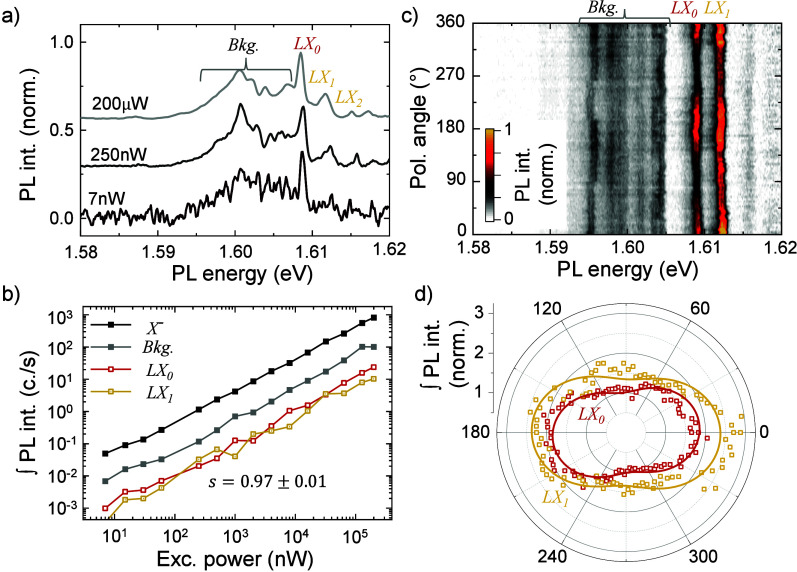
Power dependency and linearly polarized emission of 1D-confined states. (a) Trion-subtracted *LX* PL spectra in resonance under varying excitation powers: 7 nW, 250 nW, and 200 μW. (b) Integrated intensity of *X* ^–^, background (Bck.), and individual *LX*s as a function of the excitation power. The intensity follows a power-law dependence with an exponent *s* = 0.97 ± 0.05. (c) False-color plot of the Trion-subtracted resonance in *LX* PL spectra as a function of the excitation–collection linear polarization angle. (d) Polar plot of the integrated PL spectral intensity of individual *LX*s as a function of the excitation–collection linear polarization angle. Solid lines are a sinusoidal fit.

Free excitons in TMDs exhibit circular optical selection rules;^29,73,74^ however, excitons confined within a 1D channel display distinct behavior. The long-range electron–hole exchange interaction mixes exciton COM motion with *K*/*K*′ valley degrees of freedom.^32,50,75^ Motional in-plane anisotropy from the 1D trap leads to the splitting of confined exciton COM wave functions into orthogonal linearly polarized states, parallel or perpendicular to the DW. Consequently, the energy of the *n*th quantized state splits into two, one polarized parallel to the DW at energy *E*_*n*_, and a second polarized perpendicular to the DW and energy $E_{n}^{⊥}=E_{n}+\delta_{n}$ . Here, δ_*n*_ = γ*k*_*n*_, with γ the exchange–interaction coupling parameter and *k*_*n*_ the average COM wavevector.^50,75,76^

Motivated by this expectation, we performed polarization-resolved collinear excitation–collection PL spectroscopy on the localized exciton peaks at the DW. Figure 3c shows a false-color plot of the background-subtracted *LX* emission versus the excitation–emission angle relative to the DW. *LX*_0_ and *LX*_1_ show clear linear polarization parallel to the DW, with a suppressed emission perpendicular to the DW. Figure 3d shows the extracted *LX*_0_ and *LX*_1_ intensities versus angle; solid lines are sinusoidal fits. Both display ∼50% and ∼40% suppression perpendicular to the DW, consistent with previous 1D exciton observations,^32,50^ although the suppression is lower in this case. The absence of emission in the direction perpendicular to the DW aligns with previous reports of 1D states. As confinement increases, *k*_*n*_ rises, shifting perpendicularly polarized states to higher energies, thereby rendering their occupation unfavorable and suppressing their emission.^32^ In contrast, the background emission shows smaller modulation (Figure 3c) and its behavior is clearly different as the *LX*. While a precise identification of these features remains unclear and demands further understanding, they may stem from local dielectric fluctuations, or structural imperfections which modulate the 1D-trapping potential along the DW.

To explore the 1D-exciton levels and their relationship with the 2D counterpart, we conducted PLE experiments. Figure 4a shows a false-color plot of the *LX* PL spectra as a function of *E*_ex_. All features exhibit strong resonance when exciting near the *X* energy at ∼1.66 eV, indicated by the gray line, and no resonance occurs across *X* ^–^ energy. Figure 4b shows the integrated PL intensity (arrows in Figure 4a) as a function of the excitation energy. Resonant excitation of *X* increases the *LX* emission by more than 2 orders of magnitude compared to nonresonant excitation, showing that the 1D channel is efficiently excited by generating a 2D exciton population that is subsequently trapped at the DW. Notably, even when exciting at *E*_ex_ = 1.62 eV, ∼10 meV above *LX*_0_, no emission intensity increase is observed, suggesting two possible explanations: the relatively small area of the 1D channel compared to the overall laser beam size and the inherently lower oscillator strength of polarized excitons compared to *X*.

**Figure 4:**
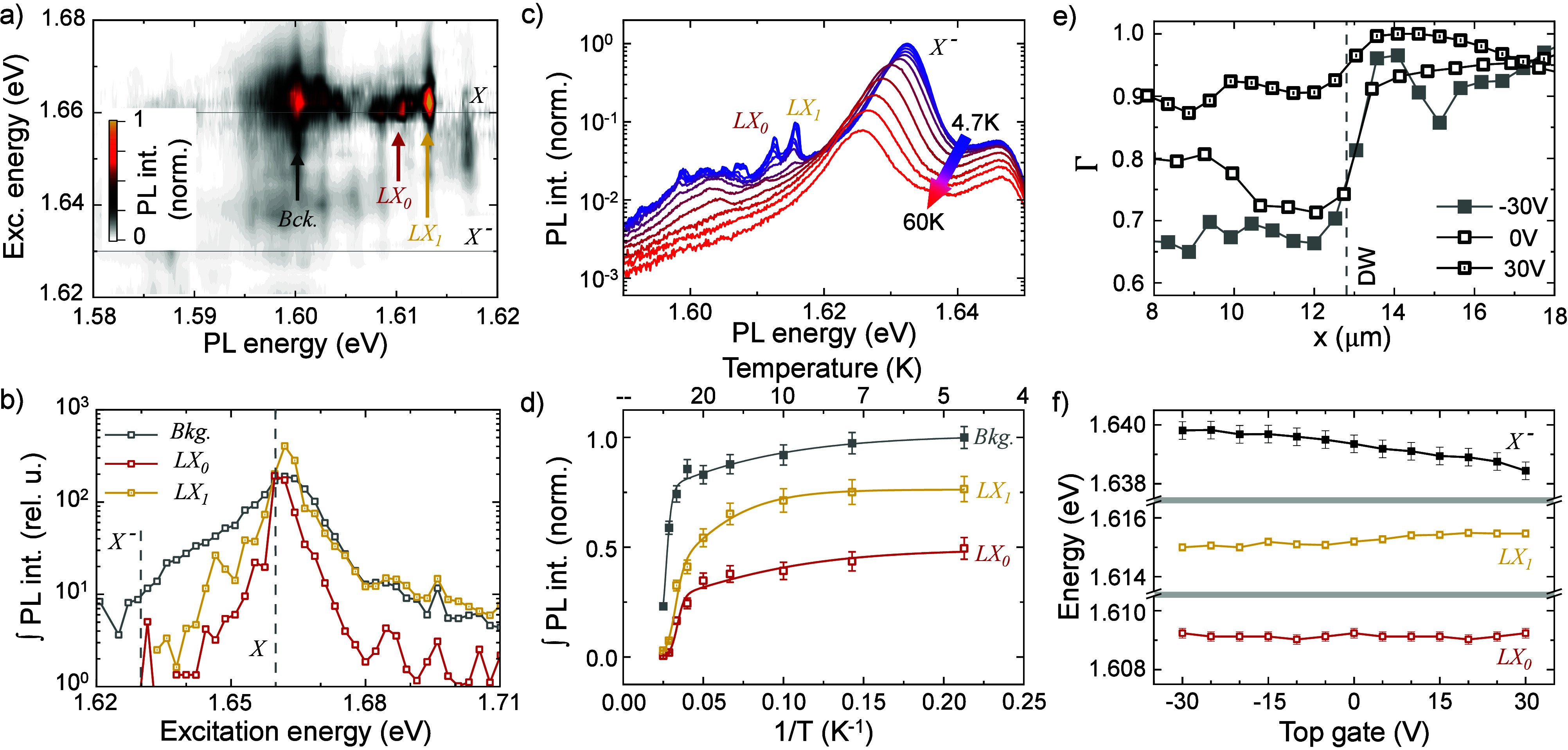
Resonance effects, temperature evolution, and free charge density effects on the 1D-confined states. (a) False-color plot of the trion-subtracted *LX* PL spectra as a function of the excitation energy revealing the resonance with *X*. (b) Integrated PL spectral intensity as a function of the excitation energy. (c) In resonance *LX*s and trion PL spectra as a function of the temperature, from 4.7 to 60 K. (d) Integrated PL intensity as a function of 1/*T*. Solid lines correspond to a modified Arrhenius fitting. (e) Line scan showing the top-gate voltage effect on the 1L-MoSe_2_ photophysics described through the parameter Γ = *I*_*X* ^–^_/(*I*_*X* ^–^_ + *I*_*X*_). (f) *X* ^–^, *LX*_0_, and *LX*_1_ spectral position as a function of the top-gate voltage.

To investigate the thermal robustness of the 1D-confined states, we performed temperature-dependent PL. Figure 4c shows PL spectra from *T* = 4.7 to 60 K, under resonant excitation at the *X* energy. The *X* ^–^ emission remains stable in intensity and spectral position up to ∼45 K. Beyond this, rising temperature causes *X* ^–^ to redshift and weaken, consistent with prior reports.^77^ In contrast, the intensities of *LX*s and the background gradually decrease, with a marked change above ∼30 K. While minor temperature-induced weakening of the electric field at the DWs may occur, it is expected to produce a gradual blueshift of the *LX* features rather than their complete suppression. In contrast, we observe that the *LX* features abruptly disappear, a behavior that we attribute to a thermal detrapping mechanism, where excitons acquire sufficient thermal energy to escape the confinement potential. Figure 4d presents the extracted intensities of *LX*_0_, *LX*_1_, and the background as functions of 1/*T*. The temperature dependence of each feature [*I*(*T*)] is fitted with a modified Arrhenius equation incorporating two activation energies, *E*_1_ and *E*_2_:

2 $$I\left(T\right)=\frac{I_{0}}{1+Ae^{-E_{1}/k_{B}T}+Be^{-E_{2}/k_{B}T}}$$

where *A* and *B* are amplitudes. The fit yields a first activation energy *E*_1_ ∼ 2 meV for all features, governing the low-temperature intensity evolution. The second activation energies are (47 ± 4), (40 ± 5), and (32 ± 5) meV for the background, *LX*_0_, and *LX*_1_, respectively. In all cases, the second energy closely matches the *X* energy difference, corroborating thermal detrapping into the 2D continuum.

Finally, we analyze the influence of free charges on the *LX* features by tuning the electron density via the top gate (Figure 1a). This effect is characterized using Γ = *I*_*X* ^–^_/(*I*_*X* ^–^_ + *I*_*X*_), where *I*_*X* ^–^_ (*I*_*X*_) is the trion (exciton) intensity. At charge neutrality, Γ → 0 as*I*_*X* ^–^_ → 0. With increasing free charge density, Γ → 1 as *I*_*X*_ → 0. Figure 4e shows line scans of Γ near the DW for three gate voltages (for further details, see SI Note VI^68^). Although 1L-MoSe_2_ is uniformly gate-biased, the effect of the charging is portrayed by the behavior of Γ on the P^–^ domain. Figure 4f shows the spectral positions of *LX*_0_, *LX*_1_, and *X* ^–^ versus gate voltage at the DW. The trion redshifts by ∼2 meV across the gate range, consistent with repulsive polaron behavior as the 2D Fermi level rises.^78^ Meanwhile, the *LX* positions remain nearly unchanged, indicating that the trapping potential is unaffected by free charge density. This contrasts with gate-induced exciton confinement,^32,50^ where the effective trapping potential results from both the Stark effect and many-body interactions and depends on the carrier density. Therefore, the confinement potential in our platform is not interaction-induced and is, consequently, insensitive to variations in the local free charge density.

We conclude by discussing the confinement potential length scales. Our calculations (Figure 1b) show that the maximum confinement energy for an encapsulated 1L-MoSe_2_ sample with 8 nm bottom hBN is −200 meV relative to *X*. However, *LX*s in this device are redshifted by only −50 meV. This suggests that the built-in electric field from the p–n homojunction at the DW diminishes *V*_Stark_, resulting in a shallower effective potential *V*_eff_. Therefore, to calculate the confined COM wave functions, we rescale *V*_Stark_ to a depth of 50 meV while preserving its profile. Solving the Schrödinger equation for *V*_eff_ using an exciton effective mass *m*_*X*_ = 1.29*m*_0_, where *m*_0_ is the electron mass,^79^ we obtain the wave functions and eigenenergies. The COM confinement, $l_{n}=\sqrt{\left\langle \psi_{n}\left|x^{2}\right|\psi_{n}\right\rangle }$ , yields l _0_ = 3.0 nm and l _1_ = 5.6 nm, with an energy separation Δ*E* = 4.5 meV. Assuming the observed *LX*_0_ and *LX*_1_ correspond to these states, the measured Δ*E* ∼ 3.5 meV, slightly position-dependent, is in good agreement with these theoretical expectations. On the other hand, using Δ*E* = ℏω within a harmonic confinement potential approximation, the estimated confinement length scales are l _0_ = 2.9 nm and l _1_ = 5.0 nm. Notably, both the harmonic approximation and the numerical estimation of *V*_eff_ yielded similar COM confinement values. More importantly, our device structure effectively decouples the 1D exciton state from the 2D counterpart by providing a potential trap that, compared to prior studies,^32,50^ is one order of magnitude higher with a ∼50% smaller COM confinement length.

## Conclusions

In summary, we leverage the large in-plane electric-field gradients at PPLN DWs to confine 1L-MoSe_2_ neutral excitons within a 1D channel. Spatially resolved μ-PL experiments reveal narrow emission lines at the DW, redshifted from the neutral 1L-MoSe_2_ exciton. These lines appear to be diffraction-limited in the direction perpendicular to the DW and extend macroscopically along it. Complementary power-dependent PL, linearly polarized PL, PLE, and temperature-dependent PL spectroscopies indicate that these lines are consistent with the formation of a 1D exciton gas at the DW. Although our design currently lacks in situ tunability of the confinement potential, the proper selection of the bottom hBN layer thickness allows manipulation of the confinement potential by up to ∼100 meV, one order of magnitude improvement over previous reports.^32,50^ This robust confinement effectively decouples the 1D exciton state from its 2D counterpart and suppresses many-body interactions with the surrounding environment. Consequently, our platform offers a compelling system for localizing and manipulating excitons in TMDs, enabling future exploration of 1D exciton dynamics and strongly as well as highly correlated phases of 1D-dipolar exciton gases.^17,18,21,23^

## Supplementary Material



## Data Availability

The data that support the findings of this study are available on request from the corresponding author.

## Notes

I-Methods, II-Determination of the electric field at the DW, III-Observation of the PPLN Raman modes superimposed on the MoSe_2_ exciton emission, IV-Estimation of the free charge density in the MoSe_2_ over different PPLN domains, V-Spatial distribution of the narrow emission lines at the DW, VI-Estimation of the strain induced over the 1L-MoSe_2_ by the DW, VII-Observation of sharp emission lines associated with 1D-confined excitons in different samples, VIII-Eigenfunctions and eigenenergies of the 1D device, and IX-Free charge density effects over *LX* (PDF)
