# Supplementary material for: Strong Quantum Confinement of 2D Excitons in an Engineered 1D Potential Induced by Proximal Ferroelectric Domain Walls
Source: Nano Lett. 2025 Aug 12;25(34):12842–50. doi: 10.1021/acs.nanolett.5c02438 (PMC12395486; doi:10.1021/acs.nanolett.5c02438)
Supplement: Supplementary file 1 [file nl5c02438_si_001.pdf]

Supplemental Information: Strong quantum confinement of 2D excitons in an engineered 1D potential induced by proximal ferroelectric domain walls

Pedro Soubelet,[1,*] Yao Tong,[1] Asier Astaburuaga Hernandez,[1]
Peirui Ji,[1] Katia Gallo,[2] Andreas V. Stier,[1] and Jonathan J. Finley[1]

[1] *Walter Schottky Institut and TUM School of Natural Sciences, Technische Universität München, Am Coulombwall 4, 85748 Garching, Germany.*
[2] *Department of Applied Physics, KTH Royal Institute of Technology, Roslagstullsbacken 21, Stockholm SE-106 91, Sweden.*

(Dated: 2025-08-04)

I. METHODS

A. Sample Fabrication

The periodically poled domains were fabricated by bulk electric field poling of commercial 500μm thick congruent z-cut $LiNbO_3$ crystals. The quality of the poling was verified optically, which allows us to visualize the domain pattern and evaluate its uniformity without any surface contamination or disruption [1]. Furthermore, reference PPLN samples fabricated in the same batch were etched for 10 min in HF acid to convert the PPLN pattern into a surface relief grating (due to the differential etching of $-z$ and $+z$ domains in HF), further confirming the successful domain inversion on both faces with a domain duty cycle close to 50% on the lithographically patterned face of the sample (originally $-z$).

Monolayers $MoSe_2$, hBN flakes and few layers graphene were obtained from commercial bulk crystals via mechanical exfoliation. We specifically selected 1L-$MoSe_2$ due to its lack of dark states below the X energy and the simplicity of its PL spectra at low temperature, therefore facilitating the identification of additional emission features that result from the 1D X confinement.

All the samples used in this work were stacked and encapsulated between thin hBN flakes using dry transfer techniques based on polycarbonate films, similar to Ref. [2].

B. Optical experiments

All experiments were conducted using a helium exchange gas cryostat, equipped with a cryogenically compatible objective to reach a diffraction-limited spot of ~ 500 nm ($100\times$ objective, NA $= 0.82$) and a temperature controller. The excitation source was a CW tunable Ti:Sa laser.

II. DETERMINATION OF THE ELECTRIC FIELD AT THE DOMAIN WALL

Figure 1a displays a schematic representation of the PPLN substrate, the bottom hBN flake and the monolayer 1L-$MoSe_2$ used to estimate the electric field at the TMD position. Figures 1b and c present the out-of-plane and the in-plane electric field at the TMD monolayer position as a function of the bottom hBN used for the sample encapsulation. The calculations were produced by finite elements assuming an hBN refractive index of $n_{hBN} = 1.8$ and an effective surface charge density on the PPLN domains of $\pm 2.7\,\mu C/\mu m^2$ as determined in our previous work [3].

Note that, by grounding the samples, the reordering of charges screens the electric field along all domains, except the in-plane electric field at the DW, where the device behaves like a nanometer scale $p-n$ homojunction generating a build in electric field that reduces V_{Stark} resulting in an effective potential V_{eff}.

* pedro.soubelet@wsi.tum.de

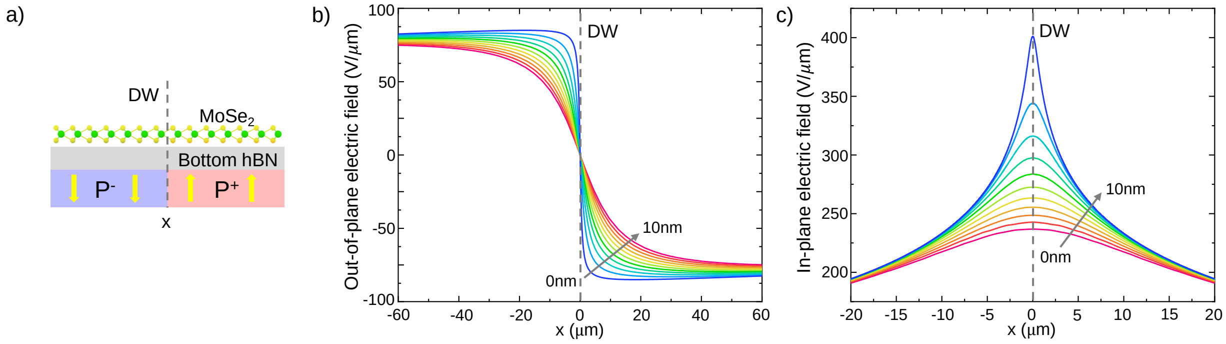

Figure 1. **Calculated electric field at the DW as a function of the bottom hBN. a)** Schematic representation of the bottom hBN flake and $MoSe_2$ monolayer used to calculate the electric field at the PPLN DW. **b)** Calculated out-of plane electric field component. **c)** Calculated in-plane electric field component.

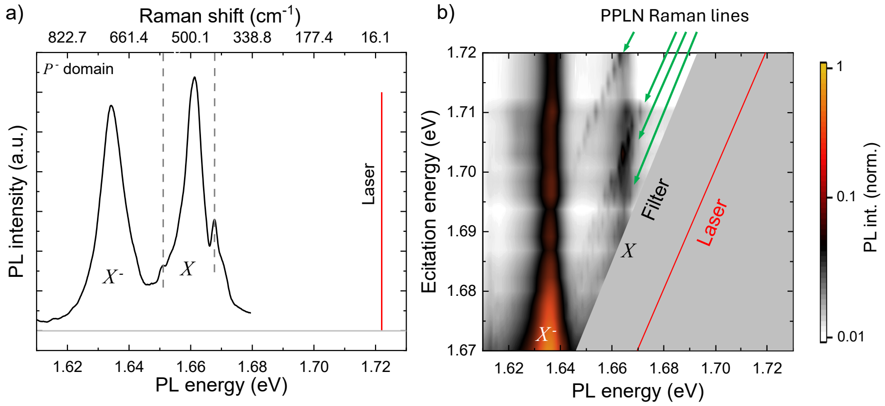

Figure 2. **Observation of Raman lines from the PPLN over the $MoSe_2$ exciton emission. a)** PL spectra recorded at the P^- domain displaying the $MoSe_2$ X and X^-. In the vicinity of the X feature, there are two additional features, at $\sim 570\,\mathrm{cm}^{-1}$ and $\sim 410\,\mathrm{cm}^{-1}$ from the laser line (red vertical line). **b)** False-colour plot of the X and X^- PL recorded at the DW as a function of the excitation energy. The sharp emission lines follow the laser energy confirming their Raman origin.

III. OBSERVATION OF PPLN RAMAN MODES SUPERIMPOSED ON THE $MOSE_2$ EXCITON EMISSION

Figure 2a presents the PL spectra of 1L-$MoSe_2$ measured at the center of the P^- domain (spot 1 in Figure 1d, main text). In this section, we analyze the sharp spectral features observed near the X emission, highlighted by dotted lines in Figure 2a, and show they are consistent with Raman modes from the PPLN substrate. These features appear redshifted with respect to the laser excitation (indicated by the red vertical line) by $\sim 570\,\mathrm{cm}^{-1}$ and $\sim 410\,\mathrm{cm}^{-1}$, which correspond well with prominent Raman-active modes previously reported for PPLN [4].

Figure 2b displays a false-color plot of the X and X^- emissions as a function of the excitation energy. These measurements were performed at the DW, where LX emissions were detected. The red line indicates the laser energy used for each measurement, while the gray-shaded area represents the spectral range excluded by optical filtering required for PLE spectroscopy. As expected, the X^- emission exhibits a pronounced resonance when the excitation energy approaches the X exciton energy, near 1.66 eV. Green arrows mark the positions of the narrow spectral lines observed near the X emission. These lines shift linearly with the laser energy, fully consistent with Raman scattering processes.

Notably, these Raman features are clearly observed only in the vicinity of the X emission and are significantly weaker in the P^+ domain, where X is strongly suppressed. Given that the PPLN substrate is optically transparent in this spectral range, there are no electronic states within the substrate to provide Raman oscillator strength. Therefore, the resonant enhancement of the Raman intensity when the Raman features coincide with the 1L-$MoSe_2$ X emission energy, indicates exciton-mediated Raman scattering.

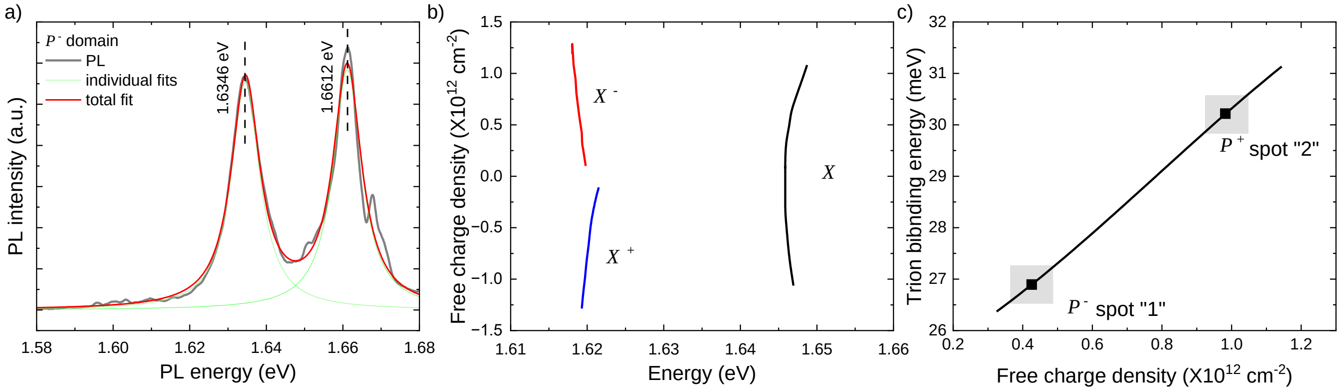

Figure 3. **Free charge density estimation.** **a)** Example of Lorentzian fits applied to the X and X^- peaks to determine their spectral positions. The spectrum shown corresponds to the P^- domain, labeled as "1" in the main text. **b)** Spectral positions of X, X^-, and X^+ as a function of free charge density, extracted from Ref. [5]. **c)** X^- binding energy derived from the data in panel b. Black square markers indicate the experimentally determined binding energies for the P^+ and P^- domains with grounded top-gate.

IV. ESTIMATION OF THE FREE CHARGE DENSITY IN THE MOSE$_2$ OVER DIFFERENT PPLN DOMAINS

In a conventional scenario, excitons can capture an additional charge from the Fermi sea to form trions [5]. However, this simple three-particle picture does not always capture the complex interactions observed between excitons and the Fermi sea [5]. When the Fermi energy approaches the trion binding energy, a more realistic description accounts for those interactions through exciton-polarons, many-body states formed when an exciton is dressed by a polarized electron-hole cloud from the Fermi sea.

Within this framework, the energy splitting between the neutral exciton and charged excitons is reinterpreted as the energy difference between repulsive and attractive polarons, respectively. Crucially, this energy splitting becomes a function of the free carrier density.

To estimate the free charge density, we analyze the trion binding energy, defined as the energy difference between the neutral exciton and the charged exciton peaks. Figure 3a illustrates the fitting procedure used to extract the emission energies of X and X^-, using Lorentzian profiles. This yields an effective binding energy of 26.7 meV. As the fitting procedure underestimates the error bar, we repeated it on ten spectra surrounding each region of interest. The resulting uncertainty, derived from the standard deviation is ± 0.3 meV.

Figure 3b shows the spectral evolution of the different $MoSe_2$ exciton complexes as a function of free charge density, extracted from Ref.[5]. While the absolute energy values are sensitive to environmental and extrinsic effects, the relative energy separation between the exciton complexes remains a robust material property. From these data, figure3c presents the trion binding energy as a function of charge density. The black squares indicate the experimentally observed binding energies at the positions labelled "1" and "2" in the main text, corresponding to the P^- and P^+ domains, respectively. Gray shaded regions represent the uncertainty in the binding energy and the resulting propagated error in the free charge density.

The estimated charge densities are therefore $n_{P^-} = (0.42 \pm 0.3) \times 10^{12}\,\mathrm{cm}^{-2}$ and $n_{P^+}(0.98 \pm 0.3) \times 10^{12}\,\mathrm{cm}^{-2}$ for the P^- and P^+ domains, respectively.

V. SPATIAL DISTRIBUTION OF THE NARROW EMISSION LINES AT THE DW

This section further characterizes the spatial distribution of the narrow emission lines associated with 1D exciton states. Figure 4a presents a false-colour map of the integrated PL intensity from 1L-$MoSe_2$ across the device, clearly revealing the alternating domains of the PPLN substrate. In figure 4b, a false-colour map of the integrated LX emission shows that it is localized along the DW, extending over approximately 12 μm across the 1L-$MoSe_2$ layer.

Figure 4c displays a false-colour plot of the PL spectrum along the DW (at $x = 12.5\,\mu$m). The emission includes the X^- feature near 1.635 eV, as well as a set of narrow LX lines appearing in the range of approximately 1.60 to 1.62 eV. These LX features typically appear in clusters of two or three lines, separated by $\sim$3 meV, and extend over 2–3 μm along the DW. In contrast, figure 4d shows a false-colour PL map taken perpendicular to the DW (at $y = 5.5\,\mu$m).

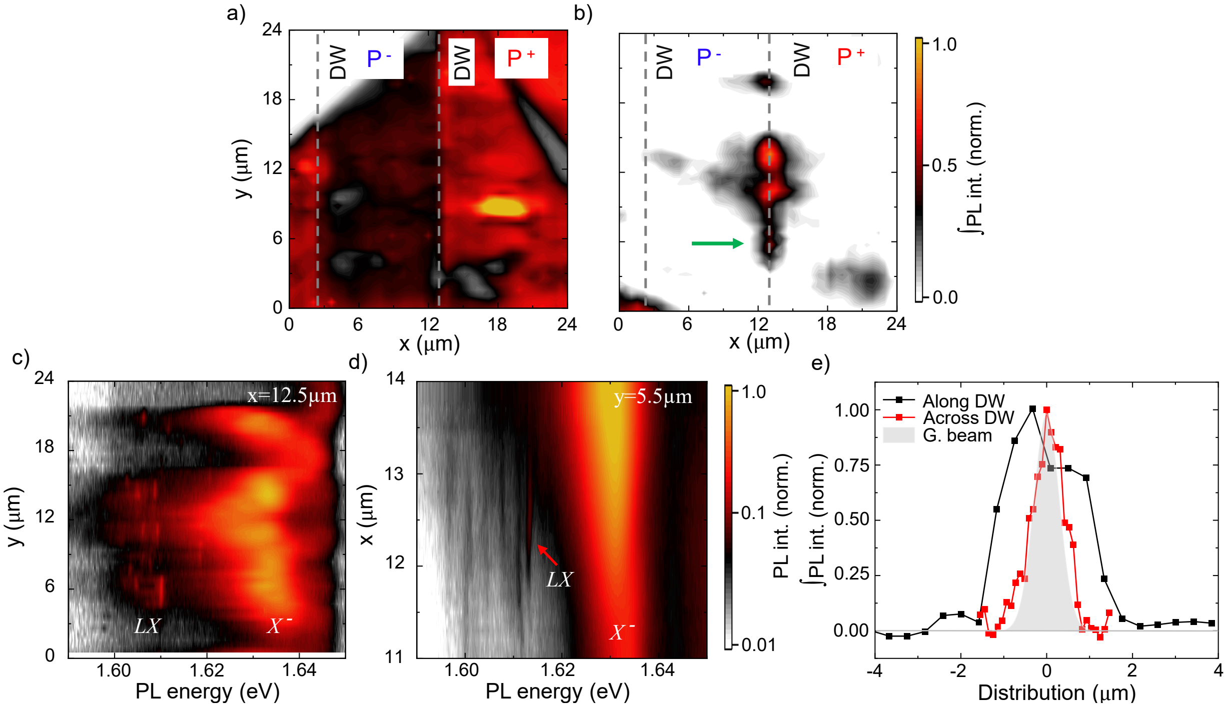

Figure 4. **Distribution of the sharp emission lines along the DW. a)** False-colour map showing the integrated PL across the sample with 8 nm bottom hBN. The spatial modulation reveals the underlying PPLN domains. **b)** False-colour map of the same sample showing the background subtracted and integrated PL intensity of the LX. **c)** False-colour plot of the LX emission along the DW (at $x = 12.5\,\mu$m). **d)** False-colour plot of the LX emission across the DW (at $y = 5.5\,\mu$m). **e)** Integrated LX emission along and across the DW of the LX peak at $x = 12.5\,\mu$m and $y = 5.5\,\mu$m (green arrow in b). The diffraction limited Gaussian spot of our μ−PL setup for light at this wavelength is displayed as gray shade.

Here, the LX emission is strictly confined to the DW, indicating its strong spatial localization.

Figure 4e quantifies the background subtracted integrated PL intensity of one of the narrow LX lines along (black curve) and across (red curve) the DW. This specific LX line, marked with a green arrow in figure 4b, is centered around $x = 12.5\,\mu$m and $y = 5.5\,\mu$m. Along the DW, the emission extends over $3\,\mu$m, while across the DW, the emission is diffraction-limited, as evidenced by its agreement with the width of the calculated Gaussian excitation spot (light gray shadow). This spatial anisotropy distinguishes the LX emission from that of point-like defects, whose PL is diffraction-limited in all directions.

VI. ESTIMATION OF THE STRAIN INDUCED OVER THE 1L-MOSE$_2$ BY THE DW

Strain in 1L-TMDs significantly modulates their bandgap, directly impacting the exciton emission energy E_X. In particular, tensile positive strain induces a redshift in the exciton emission spectrum [6, 7]. This spectral shift is quantified by the "gauge factor", which characterizes the rate of energy change per unit strain. For $MoSe_2$ under biaxial strain, the gauge factor is reported to be $a\mathrm{MoSe}_2 = -98.2\,\mathrm{meV}/\%$ [7]. While the absolute value of E_X depends on the local dielectric environment, spatial variations in E_X across the sample serve as a reliable indicator of relative strain differences. In this section we rule out the influence of strain applied over the TMD at the DW.

Figure 5a shows a false-colour map of the PL spectrum from 1L-$MoSe_2$ taken across the DW (at $y = 5.5, \mu$m, corresponding to Figure 4d). The spectrum exhibits distinct peaks corresponding to the X at ∼1.66 eV, the X^- at ∼1.63 eV, and the narrow LX emission at ∼1.61 eV. Figure 5b displays the extracted E_X obtained through Lorentzian fitting, as detailed in Supplementary Note IV. Notably, E_X displays a small variation of (0.4 ± 0.3) meV in the vicinity of the DW. However, assuming that the shift is only related to strain, it would result in a relative strain of less than $4 \times 10^{-3}\%$. This negligible strain further supports the conclusion that the LX emission is not strain-activated, and thus cannot be attributed to strain-induced defect states.

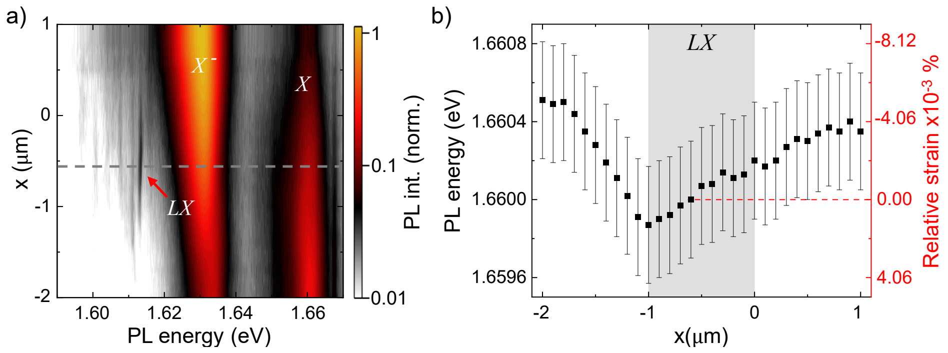

Figure 5. **Estimation of the strain induced over the sample by the DW. a)** False-colour plot of the X, X^- and LX emission across the DW. The x-scale was shifted to display the DW at $x \sim= 0\,\mu$m. **b)** Extracted exciton position across the DW. The rectangular shade marks the region in which the LX is observed. The right y-axis presents the calculated relative strain. The reference E_X was set to 1.66 eV.

VII. OBSERVATION OF SHARP EMISSION LINES ASSOCIATED TO 1D CONFINED EXCITONS IN DIFFERENT SAMPLES

The observation of sharp emission lines attributed to localized exciton states was consistently reproduced across multiple samples. The results are summarized in Figure 6, which presents data from three distinct samples. Specifically, Figs. 6a, d, and g displays the optical micrograph of each device, with the green lines marking the contour of the 1L-MoSe2 and a vertical black line marking the DW on each sample. Additionally, below each optical picture, a schematic representation shows the sample architecture. Figs. 6b, e, and h presents the false-color plot of their PL (samples in Figs. 6b, e, and h, respectively) across the DW and along the red arrows depicted in their micrographs. Finally, Figs. 6c, f, and i present, for each device in Figs. 6a, d, and g, respectively, the PL spectra at the DW. The energy scale in these figures is relative to the 2D neutral exciton.

The 1L-MoSe$_2$ flake in the sample of Fig. 6a correspond to the grounded sample presented in the main text, it was stacked with 8 nm bottom hBN, and its false color plot in Fig. 6b and its PL in c display LXs emission redshifted by $\sim$50 meV from the exciton feature. The sample in Fig. 6d has 5 nm bottom hBN and was not grounded. As a result, X and X^- spectrally shift due to the out-of-plane electric field that is not completely screened and produces Stark shift. Its false colour plot in Fig. 6e and its PL in f show the LXs redshifted by $\sim$70 meV from the exciton feature. The last sample, displayed in Fig. 6g was directly stacked on top of the PPLN and was grounded. Its false colour plot in Fig. 6h and the spectra i display the exciton and trion features and, $\sim$120 meV redshifted from the exciton, the localized states.

Our results across different samples show that it is possible to tune the 1D confinement by properly selecting the bottom hBN thickness in the device structure (see next section for details).

VIII. EIGENFUNCTIONS AND EIGENENERGIES OF THE 1D DEVICE

Using an in-plane exciton polarizability for 1L-MoSe$_2$ of $\alpha = 6.5\text{nm}^2\text{V}^{-2}$ [8] and the calculated $E_x(x)$ (see Fig. 1c), we determined the theoretical potential $V_{Stark}(x)$ presented in figure 7a. By numerically solving the Schrödinger equation for this DC Stark potential trap, we obtained the wavefunctions and eigenenergies of the system, assuming an exciton mass of $1.29m_0$, where m_0 is the electron mass [9]. The eigeneneries for the first confined states (ψ_0 to ψ_5) are plotted in figure 7b as a function of the bottom hBN thickness. The inset in Fig.7b illustrates the wavefunctions for the first confined states for a sample stacked on top of 4 nm bottom hBN.

IX. FREE CHARGE DENSITY EFFECTS OVER LX

while a comprehensive investigation of the material response lies beyond the scope of this work, we analyzed the influence of the free charge density on the LX emission by tuning the sample top gate (sample 1). To characterize the effect of the top gate on the photophysics of the 2D counterpart, we introduce the parameter $\Gamma = I_{X^-}/(I_{X^-} + I_X)$. Figure 8a and b present false-colour maps of Γ across the sample under top gate voltage of -30 V and 30 V, respectively. At -30 V, the P^- and P^+ domains exhibit distinctly different charge density, reflected by $\Gamma \simeq 0.65$ for the P^- domain

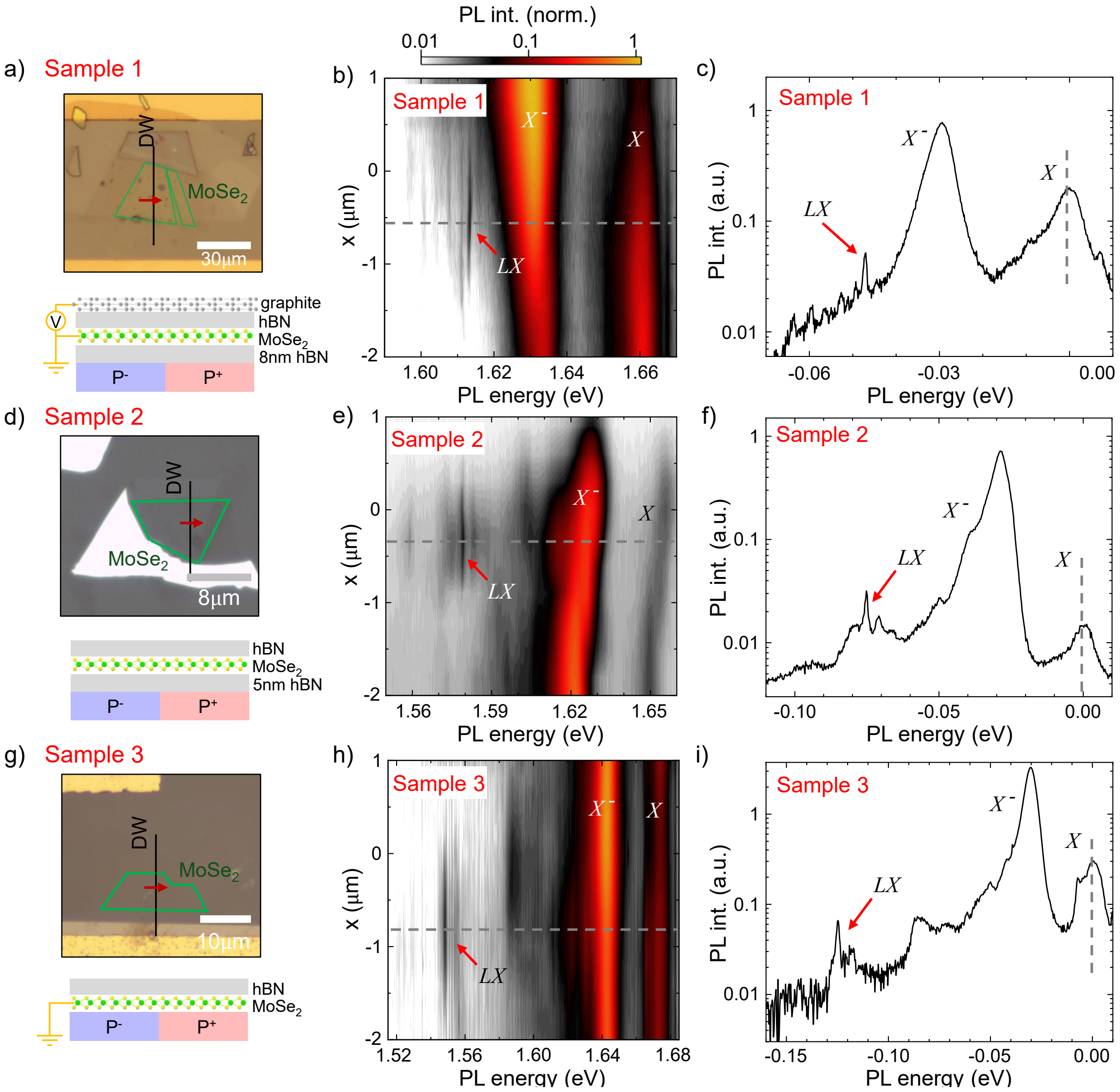

Figure 6. **PL experiments on different samples. a)** Optical micrograph and schematic representation of the 1L-$MoSe_2$ sample presented in the main text and that was stacked on top of 5 nm bottom hBN flake. **b)** False-colour plot of the integrated PL intensity across the DW for the sample in a. **c)** PL spectra at the DW for the sample in a. **d)** Optical micrograph of a 1L-$MoSe_2$ stacked on the PPLN with a 5 nm bottom hBN flake. **e)** False-colour plot of the PL intensity across the DW for the sample in d. **f)** PL spectra at the DW for the sample presented in d. **g)** Optical micrograph of a grounded 1L-$MoSe_2$ stacked directly stacked on the PPLN. **h)** False-color plot presenting the PL intensity across the DW for the sample in g. **i)** PL spectra at the DW for the sample in g. The PL spectra in c, f and i are presented on an energy scale relative to the 2D neutral exciton.

and $\Gamma \simeq 0.95$ for the P^+ domain. In contrast, the map at 30 V reveals a much more homogeneous Γ across the sample. Note that as $\Gamma \to 1$, the X emission is suppressed. The effect of the top gate is summarized in Figure 8c, which presents Γ across the DW for three different voltages. Figure 8d presents a false-colour plot of the in-resonance PL at the DW as function of the top gate voltage. Sweeping the top gate in the positive direction increases X^- intensity and induces a redshift of 2 meV, consistent with previous reports [10]. While the X emission is suppressed by increasing the charge density at positive voltages, the LXs emission increases. Figure 8e and f show the extracted intensity and spectral position for LX_0, LX_1 and X^- as a function of the top gate voltage. While LXs and X^- increase their intensity with similar proportion, the spectral position of LX_0 and LX_1 remains nearly constant, displaying a slight blueshift of ~0.5 meV.

[1] M. J. Missey, S. Russell, V. Dominic, R. G. Batchko, and K. L. Schepler, Optics Express **6**, 186 (2000).

[2] A. Castellanos-Gomez, M. Buscema, R. Molenaar, V. Singh, L. Janssen, H. S. Van Der Zant, and G. A.

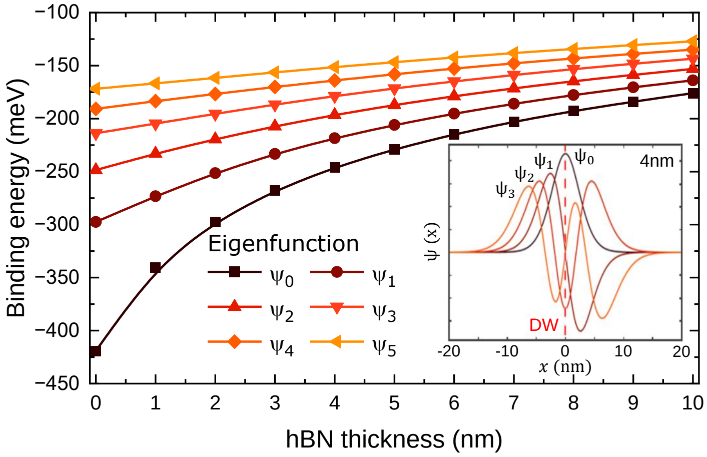

Figure 7. **Potential trap and confined states.** Theoretical calculation of the eigenenergies of the confined states as a function of the bottom hBN thickness. Inset: First wavefunctions for a sample stacked on 4 nm hBN.

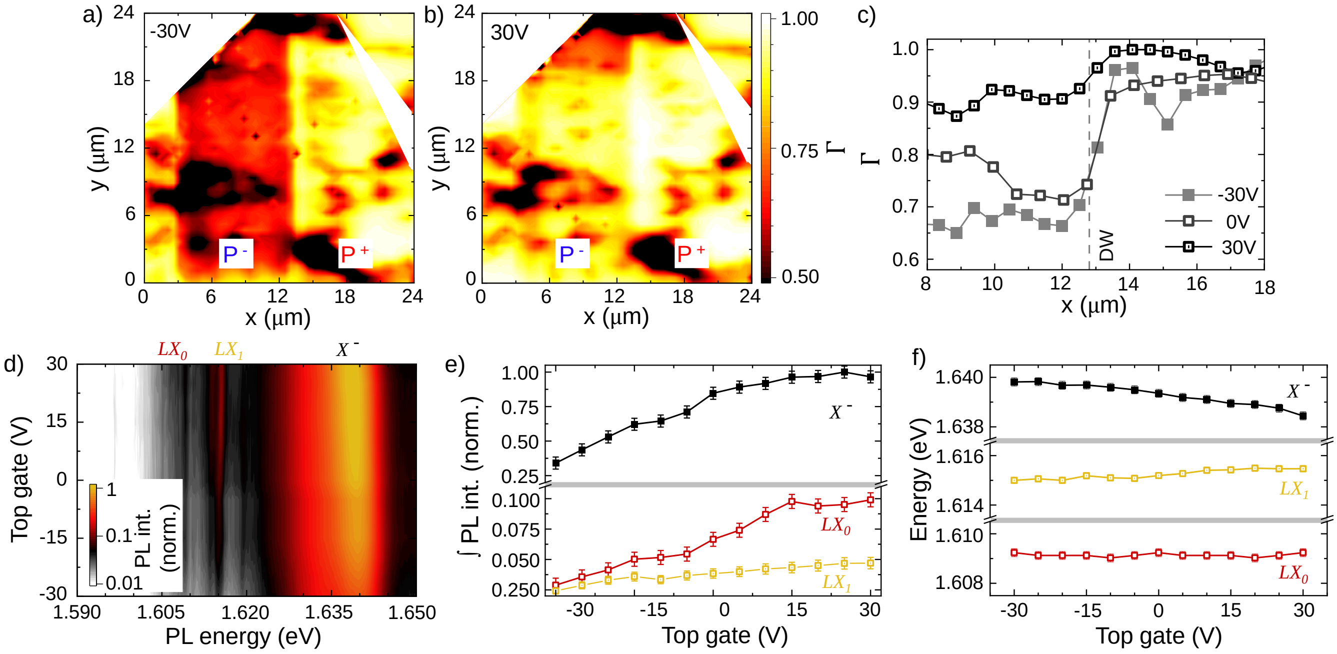

Figure 8. **Electronic landscape effect over LXs. a** and **b)** False colour maps showing the top gate effect over the $MoSe_2$ photophysics through the parameter $\Gamma = I_{X^-}/(I_{X^-} + I_X)$. **c)** Γ modulation across the DW for different top gate voltages. **d)** False colour plot of the LX and trion PL spectra in resonance as a function of the top gate. **e)** X^-, LX_0 and LX_1 intensity as a function of the top gate voltage. **f)** X^-, LX_0 and LX_1 spectral position as a function of the top gate.

Steele, 2D Materials **1**, 011002 (2014).

[3] P. Soubelet, J. Klein, J. Wierzbowski, R. Silvioli, F. Sigger, A. V. Stier, K. Gallo, and J. J. Finley, Nano Letters **21**, 959 (2021).

[4] G. F. Nataf, M. Guennou, A. Haußmann, N. Barrett, and J. Kreisel, physica status solidi (RRL)–Rapid Research Letters **10**, 222 (2016).

[5] E. Liu, J. van Baren, Z. Lu, T. Taniguchi, K. Watanabe, D. Smirnov, Y.-C. Chang, and C. H. Lui, Nature communications **12**, 6131 (2021).

[6] Z. An, P. Soubelet, Y. Zhumagulov, M. Zopf, A. Delhomme, C. Qian, P. E. Faria Junior, J. Fabian, X. Cao, J. Yang, *et al.*, Physical Review B **108**, L041404 (2023).

[7] K. Zollner, P. E. Faria Junior, and J. Fabian, Physical Review B **100**, 195126 (2019).

[8] L. Cavalcante, D. R. da Costa, G. Farias, D. Reichman, and A. Chaves, Physical Review B **98**, 245309 (2018).

[9] L. Li, H. Yang, and P. Yang, Journal of Colloid and Interface Science **650**, 1312 (2023).

[10] J. S. Ross, S. Wu, H. Yu, N. J. Ghimire, A. M. Jones, G. Aivazian, J. Yan, D. G. Mandrus, D. Xiao, W. Yao,

et al., Nature communications **4**, 1474 (2013).
